# The genome sequence of an opomyzid fly, *Geomyza tripunctata* Fallen, 1823 (Diptera: Opomyzidae)

**DOI:** 10.12688/wellcomeopenres.27071.1

**Published:** 2026-07-20

**Authors:** Liam M. Crowley, Steven Falk, Olga Sivell, University of Oxford and Wytham Woods Genome Acquisition Lab, Darwin Tree of Life Barcoding Collective, Wellcome Sanger Institute Tree of Life Management, Samples and Laboratory team, Wellcome Sanger Institute Scientific Operations: Sequencing Operations, Wellcome Sanger Institute Tree of Life Core Informatics team, Tree of Life Core Informatics collective, Darwin Tree of Life Consortium

**Affiliations:** 1 University of Oxford, Oxford, England, UK; 2 Independent researcher, Kenilworth, Warwickshire, England, UK; 3 Natural History Museum, London, England, UK

**Keywords:** Geomyza tripunctata, opomyzid fly, Cereal Fly, genome sequence, chromosomal, Diptera

## Abstract

We present a genome assembly from an individual female *Geomyza tripunctata* (opomyzid fly; Arthropoda; Insecta; Diptera; Opomyzidae). The genome sequence has a total length of 223.30 megabases. Most of the assembly (99.67%) is scaffolded into 3 chromosomal pseudomolecules. The mitochondrial genome has also been assembled, with a length of 16.03 kilobases. This assembly was generated as part of the Darwin Tree of Life project, which produces genomes for eukaryotic species found in Britain and Ireland.

## Species taxonomy

Eukaryota; Opisthokonta; Metazoa; Eumetazoa; Bilateria; Protostomia; Ecdysozoa; Panarthropoda; Arthropoda; Mandibulata; Pancrustacea; Altocrustacea; Allotriocarida; Hexapoda; Insecta; Dicondylia; Pterygota; Neoptera; Eumetabola; Endopterygota; Aparaglossata; Panorpida; Diptera; Brachycera; Muscomorpha; Eremoneura; Cyclorrhapha; Schizophora; Acalyptratae; Opomyzoidea; Opomyzidae; *Geomyza*; *Geomyza tripunctata* Fallen, 1823 (NCBI:txid500269).

## Background

Opomyzidae are a small family of flies in the monophyletic superfamily Opomyzoidea, with Anthomyzidae as their sister-group ( Griffiths, 1972; Roháček, 1998). Worldwide there are approximately 55 recognized species in three genera. In Britain this family is represented by 16 species in the genera *Opomyza* Fallén, 1820 (six species) and *Geomyza* Fallén, 1810 (ten species) ( Chandler, 2025; Drake, 1993). Here we present a first complete genome of an Opomyzidae species, *Geomyza tripunctata* Fallén, 1823.

*Geomyza tripunctata* is a small, distinctive fly with a body length 2.5–3.75 mm ( Drake, 1993). The thorax is usually dark, greyish, but it can also be orange; the abdomen is black, legs mostly yellowish. The wing pattern is characteristic, consisting of three large black spots (hence the name *tripunctata*), and usually with an additional dark cloud at base near radial stem RS ( Clements, 2020; Drake, 1993). There are three species in Britain with a similar wing pattern, however, the anepimeron of *Geomyza majuscula* (Loew, 1864) has only 3 to 5 weak setulae, while *G. tripunctata* has one strong seta in addition to 1 to 2 short setulae in the upper hind corner of the anepimeron. The third species, *G. nartshukae* Carles-Tolrá, 1993 can be separated from *G. tripunctata* using genitalia characters. *G. nartshukae* is a rare, southern species in Britain ( Clements, 2020).

The egg of *G. tripunctata* is white, fusiform, with longitudinal grooves and ridges, and is covered in minute papillae; it was described and figured by Thomas (1938) and Ferrar (1987). The larva measures up to 8 mm and is creamy in colour; it was described by Nye (1958) and Ferrar (1987). The puparium is dark brown, with protruding larval prothoracic spiracles and a deeply wrinkled cuticle at the posterior end ( Balachowsky & Mesnil, 1935; Ferrar, 1987; Thomas, 1938; van Zuijlen, 2021). The larvae develop in grasses, mostly *Lolium* Linnaeus 1753 spp. and pupate within the grass stem ( Drake, 1993; Nye, 1958; Thomas, 1938). There are three larval instars. This species overwinters mainly as a third instar larva ( Drake, 1993). The adults can be encountered all year round, but mostly from March to November ( Drake, 1993; GBIF Secretariat, 2025). The larvae can be hosts to the braconid wasps *Chasmodon apterus* (Nees, 1812) and *Phaenocarpa livida* (Haliday, 1838) and pteromalid *Stenomalina* Ghesquière, 1946 species ( Moore *et al.*, 1986; Thomas, 1938).

*Geomyza tripunctata* is a Holarctic species, very common and widely distributed, occurring in all types of grassy habitats ( Clements, 2020; Drake, 1993; GBIF Secretariat, 2025; van Zuijlen, 2021). It was introduced to North America most likely by human activity in the 1980s and has since expanded its range rapidly ( Wheeler *et al.*, 1999). *G. tripunctata* is a pest species of ley and maize ( Brunel & Crespy, 1974; Leclant, 1977; Moore & Clements, 1984).

This high-quality genome of *Geomyza tripunctata* was sequenced from a single male (SAMEA114644871) from Hitchcopse Pit, Oxfordshire, England. The genome of *G. tripunctata* presented here was sequenced as part of the Darwin Tree of Life Project, a collaborative effort to sequence all named eukaryotic species in the Atlantic Archipelago of Britain and Ireland. It will aid research on phylogeny of Acalyptratae, in particular Opomyzoidea and biology and taxonomy of the species.

## Methods

### Sample acquisition and DNA barcoding

**Figure 1.**
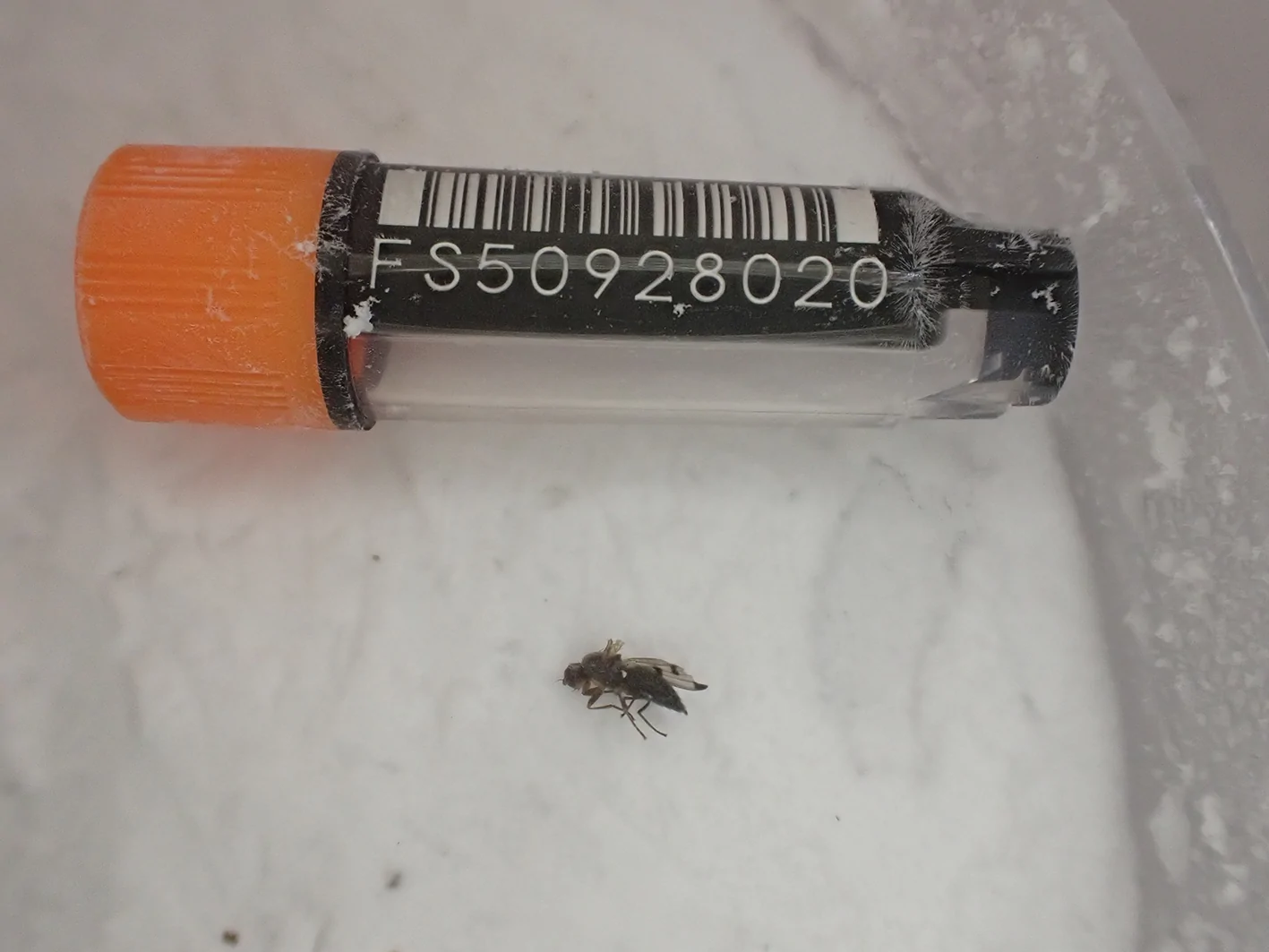
Photograph of the *Geomyza tripunctata* (idGeoTrip2) specimen used for genome sequencing.

Two adult female *Geomyza tripunctata* specimens were used: the genome-sequencing specimen (specimen ID Ox003835, ToLID idGeoTrip2; Figure 1) and the Hi-C specimen (specimen ID Ox002006, ToLID idGeoTrip1). The genome-sequencing specimen was collected from Hitchcopse Pit, Oxfordshire, UK (latitude 51.694, longitude −−1.347) on 2023-05-23 by Liam Crowley and Steven Falk and identified by Steven Falk. The Hi-C specimen was collected from Wytham Woods, Oxfordshire, UK (latitude 51.764, longitude −1.337) on 2021-12-16 by Liam Crowley, who also identified it.

The initial identification was verified by an additional DNA barcoding process according to the framework developed by Twyford *et al.* (2024). A small sample was dissected from the specimen and stored in ethanol, while the remaining parts were shipped on dry ice to the Wellcome Sanger Institute (WSI) (see the protocol). The tissue was lysed, the COI marker region was amplified by PCR, and amplicons were sequenced and compared to the BOLD database, confirming the species identification ( Crowley *et al.*, 2023). Following whole genome sequence generation, the relevant DNA barcode region was also used alongside the initial barcoding data for sample tracking at the WSI ( Twyford *et al.*, 2024). The standard operating procedures for Darwin Tree of Life barcoding are available on protocols.io.

### Nucleic acid extraction

Detailed protocols for nucleic acid extraction developed at the Wellcome Sanger Institute (WSI) Tree of Life Core Laboratory are available on protocols.io ( Howard *et al.*, 2025). The idGeoTrip2 specimen was weighed and triaged to determine the appropriate extraction protocol. For high molecular weight (HMW) DNA extraction, 0.100 mg of tissue from the whole organism was used. Tissue from the whole organism was homogenised by powermashing using a PowerMasher II tissue disruptor. HMW DNA was extracted using the Automated MagAttract v2 protocol. DNA was sheared into an average fragment size of 12–20 kb following the Megaruptor®3 for LI PacBio protocol. Sheared DNA was purified by automated SPRI (solid-phase reversible immobilisation). The concentration of the sheared and purified DNA was assessed using a Nanodrop spectrophotometer and Qubit Fluorometer using the Qubit dsDNA High Sensitivity Assay kit. Fragment size distribution was evaluated by running the sample on the FemtoPulse system. For this sample, the final post-shearing DNA had a Qubit concentration of 12.1 ng/μL, with a fragment size of 17.0 kb.

### PacBio HiFi library preparation and sequencing

Library preparation and sequencing were performed at the WSI Scientific Operations core. Libraries were prepared using the SMRTbell Prep Kit 3.0 (Pacific Biosciences, California, USA), following the manufacturer’s instructions. The kit includes reagents for end repair/A-tailing, adapter ligation, post-ligation SMRTbell bead clean-up, and nuclease treatment. Size selection and clean-up were performed using diluted AMPure PB beads (Pacific Biosciences). DNA concentration was quantified using a Qubit Fluorometer v4.0 (ThermoFisher Scientific) and the Qubit 1X dsDNA HS assay kit. Final library fragment size was assessed with the Agilent Femto Pulse Automated Pulsed Field CE Instrument (Agilent Technologies) using the gDNA 55 kb BAC analysis kit.

The sample was sequenced on a Revio instrument (Pacific Biosciences, California, USA). Prepared libraries selected for multiplexing were pooled based on genome size, library molarity, and the intended plex level. Primers were annealed and polymerases were bound to generate circularised complexes, following the manufacturer’s instructions. Complexes were purified using SMRTbell beads, diluted to the Revio loading concentration, and spiked with a Revio sequencing internal control. The pooled libraries were sequenced on a Revio 25 M Plex SMRT cell in a 4-plex run. SMRT Link software (Pacific Biosciences), a web-based workflow manager, was used to configure and monitor the run and to carry out primary and secondary data analysis.

### Hi-C

***Sample preparation and crosslinking***

The Hi-C sample was prepared from 20–50 mg of frozen whole organism tissue of the idGeoTrip1 sample using the Arima-HiC v2 kit (Arima Genomics). Following the manufacturer’s instructions, tissue was fixed and DNA crosslinked using TC buffer to a final formaldehyde concentration of 2%. The tissue was homogenised using the Diagnocine Power Masher-II. Crosslinked DNA was digested with a restriction enzyme master mix, biotinylated, and ligated. Clean-up was performed with SPRISelect beads before library preparation. DNA concentration was measured with the Qubit Fluorometer (Thermo Fisher Scientific) and Qubit HS Assay Kit. The biotinylation percentage was estimated using the Arima-HiC v2 QC beads.

***Hi-C library preparation and sequencing***

Biotinylated DNA constructs were fragmented using a Covaris E220 sonicator and size selected to 400–600 bp using SPRISelect beads. DNA was enriched with Arima-HiC v2 kit Enrichment beads. End repair, A-tailing, and adapter ligation were carried out with the NEBNext Ultra II DNA Library Prep Kit (New England Biolabs), following a modified protocol where library preparation occurs while DNA remains bound to the Enrichment beads. Library amplification was performed using KAPA HiFi HotStart mix and a custom Unique Dual Index (UDI) barcode set (Integrated DNA Technologies). Depending on sample concentration and biotinylation percentage determined at the crosslinking stage, libraries were amplified with 10–16 PCR cycles. Post-PCR clean-up was performed with SPRISelect beads. Libraries were quantified using the AccuClear Ultra High Sensitivity dsDNA Standards Assay Kit (Biotium) and a FLUOstar Omega plate reader (BMG Labtech).

Prior to sequencing, libraries were normalised to 10 ng/μL. Normalised libraries were quantified again to create equimolar and/or weighted 2.8 nM pools. Pool concentrations were checked using the Agilent 4200 TapeStation (Agilent) with High Sensitivity D500 reagents before sequencing. Sequencing was performed using paired-end 150 bp reads on the Illumina NovaSeq 6000.

### Genome assembly

Prior to assembly of the PacBio HiFi reads, a database of *k*-mer counts ( *k* = 31) was generated from the filtered reads using FastK. GenomeScope2 ( Ranallo-Benavidez *et al.*, 2020) was used to analyse the *k*-mer frequency distributions, providing estimates of genome size, heterozygosity, and repeat content.

The HiFi reads were assembled using Hifiasm ( Cheng *et al.*, 2021) with the --primary option. The Hi-C reads ( Rao *et al.*, 2014) were mapped to the primary contigs using bwa-mem2 ( Vasimuddin *et al.*, 2019), and the contigs were scaffolded in YaHS ( Zhou *et al.*, 2023) with the --break option for handling potential misassemblies. The scaffolded assemblies were evaluated using Gfastats ( Formenti *et al.*, 2022), BUSCO ( Manni *et al.*, 2021) and MerquryFK ( Rhie *et al.*, 2020).

The mitochondrial genome was assembled using MitoHiFi ( Uliano-Silva *et al.*, 2023). The MitoHiFi reference was the mitochondrial genome of *Liriomyza chinensis* (NC_041083.1).

### Assembly curation

The assembly was decontaminated using the Assembly Screen for Cobionts and Contaminants ( ASCC) pipeline. TreeVal was used to generate the flat files and maps for use in curation. Manual curation was conducted primarily in PretextView and HiGlass ( Kerpedjiev *et al.*, 2018). Scaffolds were visually inspected and corrected as described by Howe *et al*. (2021). Manual corrections included 33 breaks and 94 joins. This reduced the scaffold count by 48.0% and increased the scaffold N50 by 4.8%. The curation process is described at sanger-tol/curation-resources . PretextSnapshot was used to generate a Hi-C contact map of the final assembly.

### Assembly quality assessment

The MerquryFK tool ( Rhie *et al.*, 2020) was run in a Singularity container ( Kurtzer *et al.*, 2017) to evaluate *k*-mer completeness and assembly quality for the primary and alternate haplotypes using the *k*-mer database ( *k* = 31) computed prior to genome assembly. The analysis outputs included assembly QV scores and completeness statistics.

The genome was analysed using the BlobToolKit pipeline, a Nextflow implementation of the earlier Snakemake version ( Challis *et al.*, 2020). The pipeline aligns PacBio reads using minimap2 ( Li, 2018) and SAMtools ( Danecek *et al.*, 2021) to generate coverage tracks. It runs BUSCO ( Manni *et al.*, 2021) using lineages identified from the NCBI Taxonomy ( Schoch *et al.*, 2020). For the three domain-level lineages, BUSCO genes are aligned to the UniProt Reference Proteomes database ( Bateman *et al.*, 2023) using DIAMOND blastp ( Buchfink *et al.*, 2021). The genome is divided into chunks based on the density of BUSCO genes from the closest taxonomic lineage, and each chunk is aligned to the UniProt Reference Proteomes database with DIAMOND blastx. Sequences without hits are chunked using seqtk and aligned to the NT database with blastn ( Altschul *et al.*, 1990). The BlobToolKit suite consolidates all outputs into a blobdir for visualisation. The BlobToolKit pipeline was developed using nf-core tooling ( Ewels *et al.*, 2020) and MultiQC ( Ewels *et al.*, 2016), with containerisation through Docker ( Merkel, 2014) and Singularity ( Kurtzer *et al.*, 2017).

## Genome sequence report

### Sequence data

**Figure 2.**
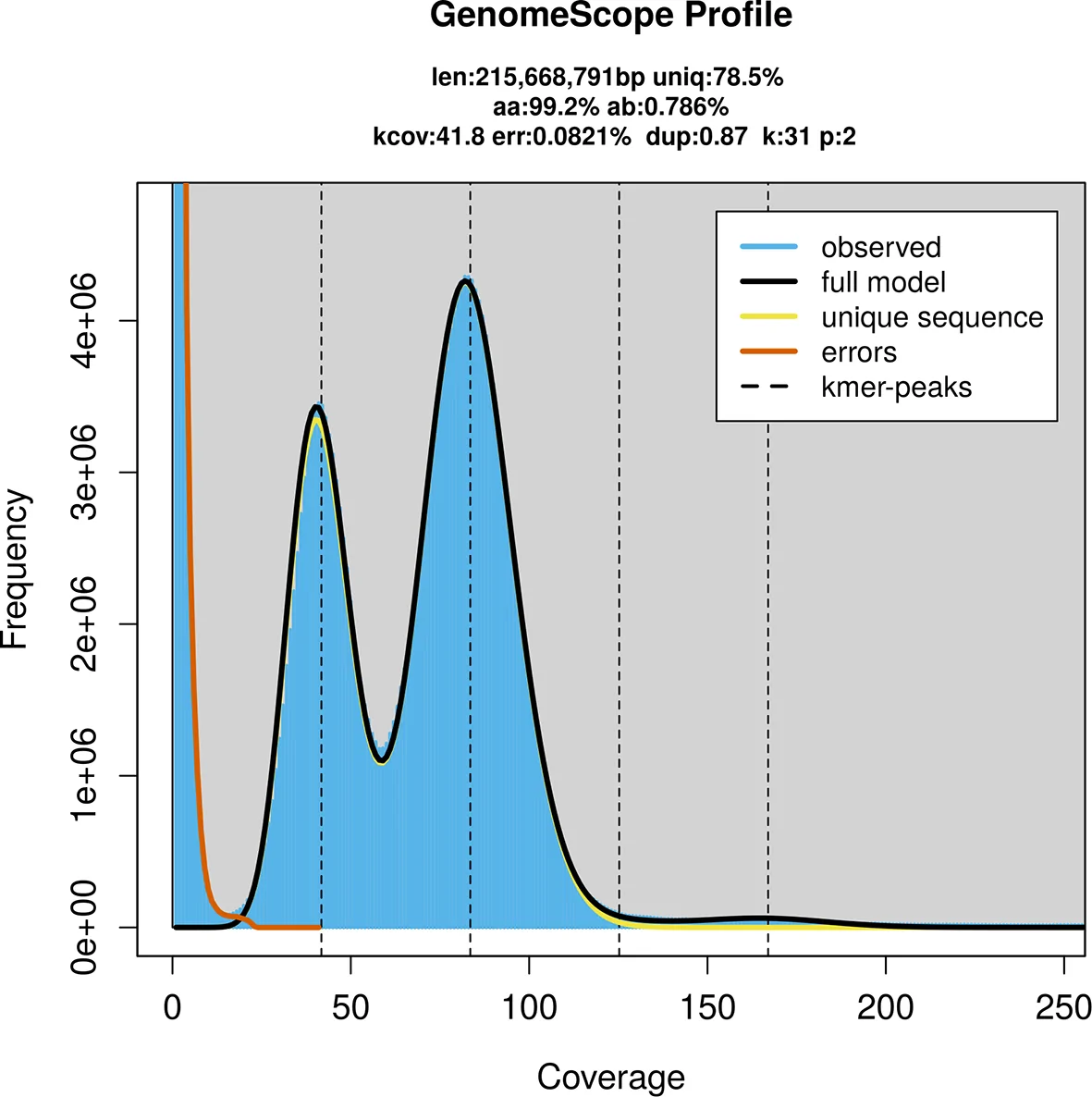
Frequency distribution of *k*-mers generated using GenomeScope2. The plot shows observed and modelled *k*-mer spectra, providing estimates of genome size, heterozygosity, and repeat content based on unassembled sequencing reads.

PacBio sequencing of the *Geomyza tripunctata* specimen generated 18.53 Gb (gigabases) from 1.90 million reads, which were used to assemble the genome. GenomeScope2.0 analysis estimated the haploid genome size at 215.81 Mb, with a heterozygosity of 0.79% and repeat content of 21.47% ( Figure 2). These estimates guided expectations for the assembly. Based on the estimated genome size, the sequencing data provided approximately 84× coverage. Hi-C sequencing produced 90.23 Gb from 298.79 million reads, which were used to scaffold the assembly. Table 1 summarises the specimen and sequencing details.

**Table 1.** Specimen and sequencing data for *Geomyza tripunctata* (BioProject PRJEB85202).

Platform	PacBio HiFi	Hi-C
**ToLID**	idGeoTrip2	idGeoTrip1
**Specimen ID**	Ox003835	Ox002006
**BioSample (source individual)**	SAMEA114644871	SAMEA110451543
**BioSample (tissue)**	SAMEA114645539	SAMEA110451729
**Tissue**	whole organism	whole organism
**Instrument**	Revio	Illumina NovaSeq 6000
**Run accessions**	ERR14209184	ERR14224660
**Read count total**	1.90 million reads	298.79 million read pairs
**Base count total**	18.53 Gb	90.23 Gb

### Assembly statistics

The primary haplotype was assembled, and contigs corresponding to an alternate haplotype were also deposited in INSDC databases. The final assembly has a total length of 223.30 Mb in 12 scaffolds, with 115 gaps, and a scaffold N50 of 69.93 Mb ( Table 2).

**Table 2.** Genome assembly data for *Geomyza tripunctata.*

Genome assembly	Primary assembly
**Assembly name**	idGeoTrip2.1
**Assembly accession**	GCA_965234165.1
**Alternate haplotype accession**	GCA_965234075.1
**Assembly level**	chromosome
**Span (Mb)**	223.30
**Number of chromosomes**	3
**Number of contigs**	127
**Contig N50**	3.58 Mb
**Number of scaffolds**	12
**Scaffold N50**	69.93 Mb
**Organelles**	Mitochondrial genome: 16.03 kb

The scaffold count follows the NCBI assembly statistics and excludes organelle assemblies, which are reported separately where present.

**Figure 3.**
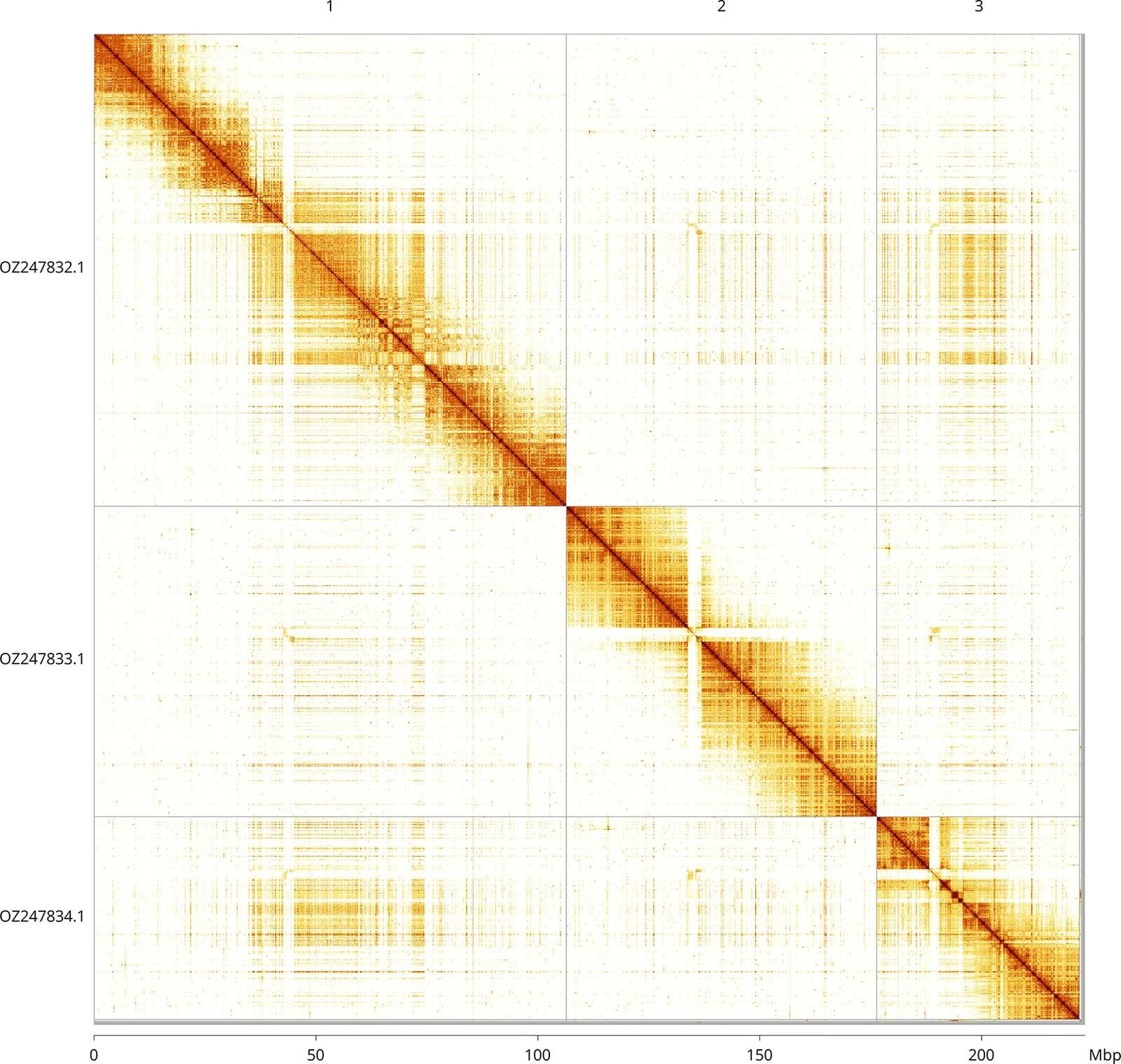
Hi-C contact map of the *Geomyza tripunctata* genome assembly. Assembled chromosomes are shown in order of size and labelled along the axes, with a megabase scale shown below. The plot was generated using PretextSnapshot.

Most of the assembly sequence (99.67%) was assigned to 3 chromosomal-level scaffolds. These chromosome- level scaffolds, confirmed by Hi-C data, are named according to size ( Figure 3; Table 3). We did not assign the sex chromosome(s) as sequence data from the heterogametic sex was not available and homology is unreliable for sex chromosome identification in Diptera due to frequent sex chromosome turnover ( Vicoso & Bachtrog, 2015). The order and orientation of the contigs are uncertain in the following regions: chromosome 1 (42 683–45 130 kbp), chromosome 2 (27 486–30 485 kbp), and chromosome 3 (16 753–19 329 kbp).

**Table 3.** Chromosomal pseudomolecules in the primary genome assembly of *Geomyza tripunctata* idGeoTrip2.

INSDC accession	Molecule	Length (Mb)	GC%
OZ247832.1	1	106.48	45.50
OZ247833.1	2	69.93	45.50
OZ247834.1	3	46.15	43

The mitochondrial genome was also assembled (length 16.03 kb, OZ247835.1). This sequence is included as a contig in the multifasta file of the genome submission and as a standalone record.

### Assembly quality metrics

**Figure 4.**
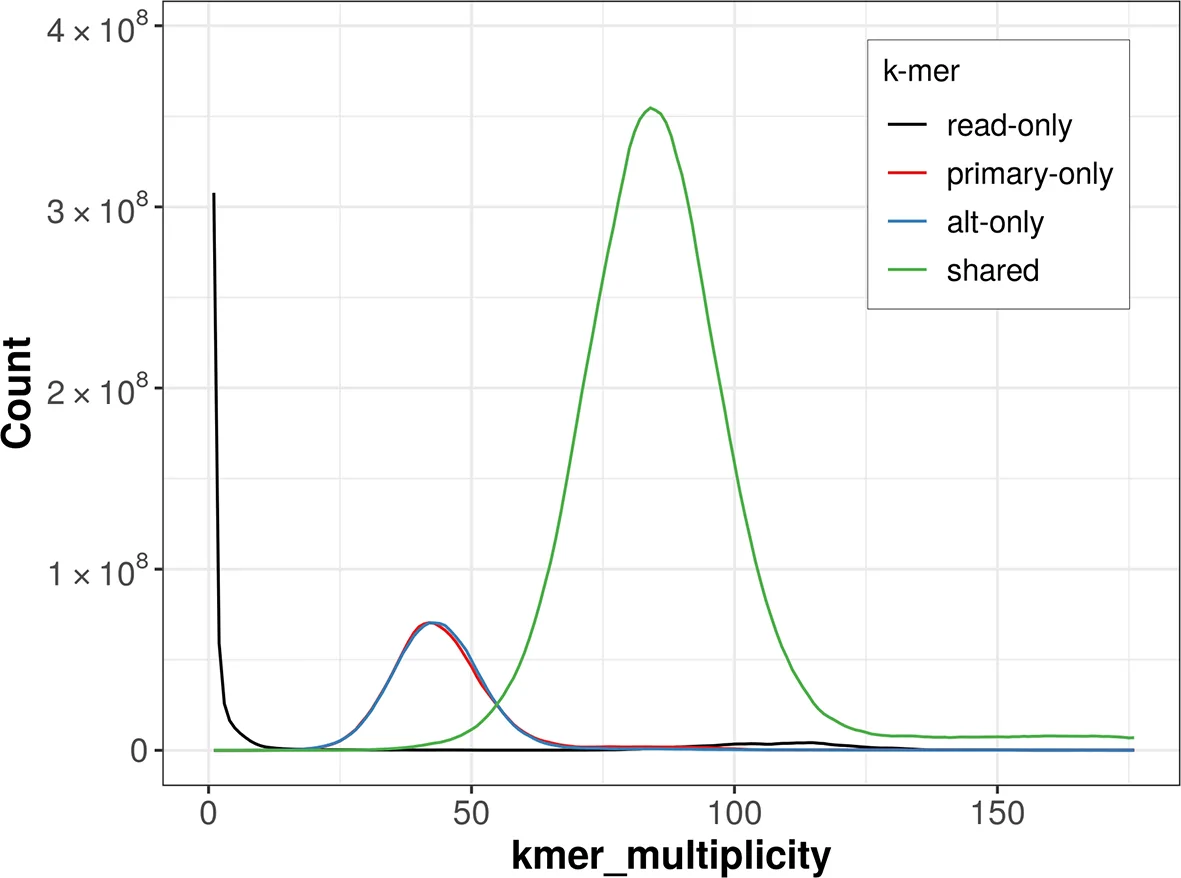
Evaluation of *k*-mer completeness using MerquryFK. This plot illustrates the recovery of *k*-mers from the original read data in the final assemblies. The horizontal axis represents *k*-mer multiplicity, and the vertical axis shows the number of *k*-mers. The black curve represents *k*-mers that appear in the reads but are not assembled. The green curve corresponds to *k*-mers shared by both haplotypes, and the red and blue curves show *k*-mers found only in one of the haplotypes.

The combined primary and alternate assemblies achieve an estimated QV of 65.9. The *k*-mer completeness is 82.85% for the primary assembly, 82.80% for the alternate haplotype, and 99.17% for the combined assemblies ( Figure 4).

**Figure 5.**
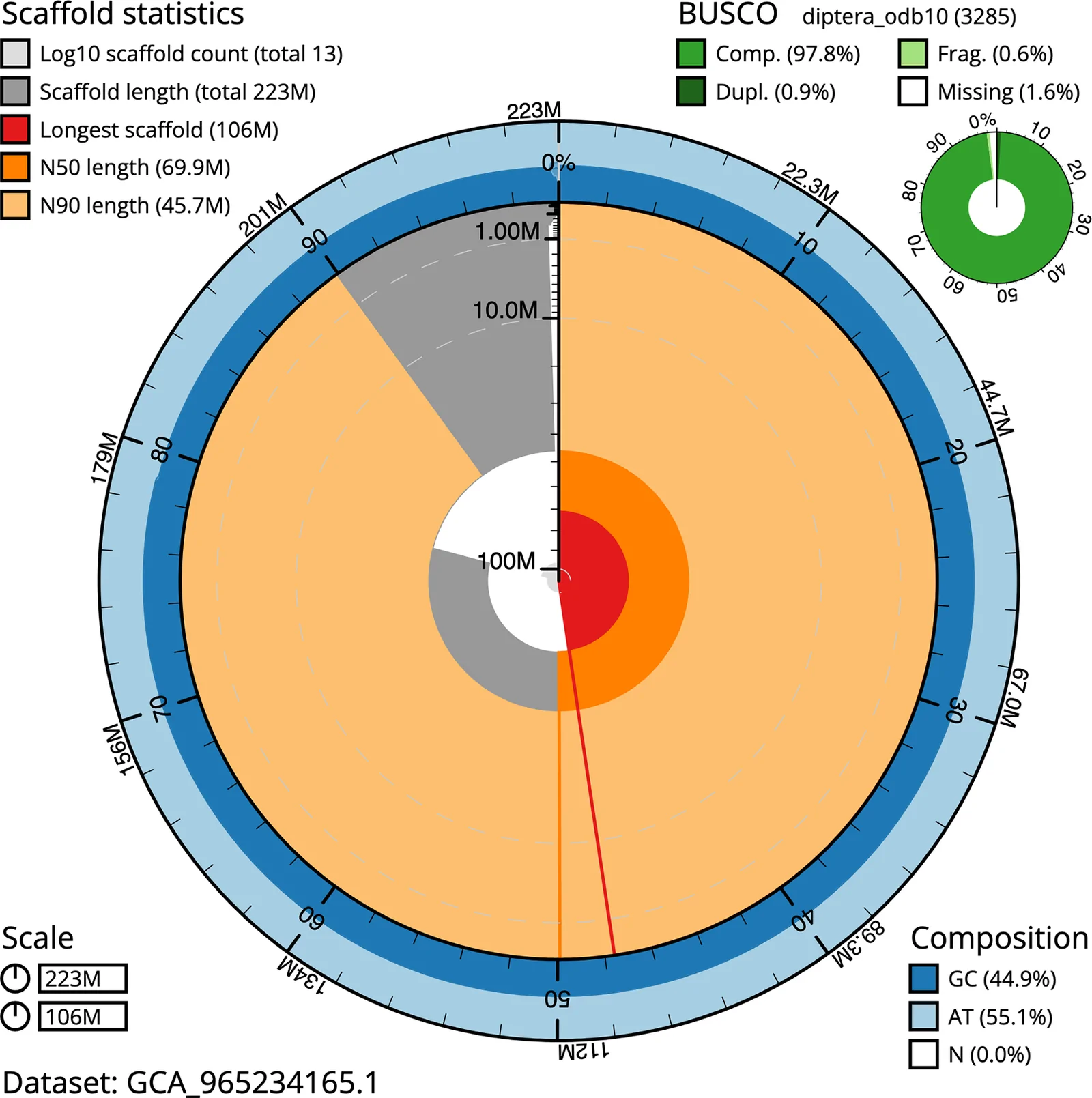
Assembly metrics for idGeoTrip2.1. The BlobToolKit snail plot provides an overview of assembly metrics and BUSCO gene completeness. The circumference represents the length of the whole genome sequence, and the main plot is divided into 1 000 bins around the circumference. The outermost blue tracks display the distribution of GC, AT, and N percentages across the bins. Scaffolds are arranged clockwise from longest to shortest and are depicted in dark grey. The longest scaffold is indicated by the red arc, and the deeper orange and pale orange arcs represent the N50 and N90 lengths. A light grey spiral at the centre shows the cumulative scaffold count on a logarithmic scale. A summary of complete, fragmented, duplicated, and missing BUSCO genes in the diptera_odb10 set is presented at the top right. An interactive version of this figure can be accessed on the BlobToolKit viewer.

**Figure 6.**
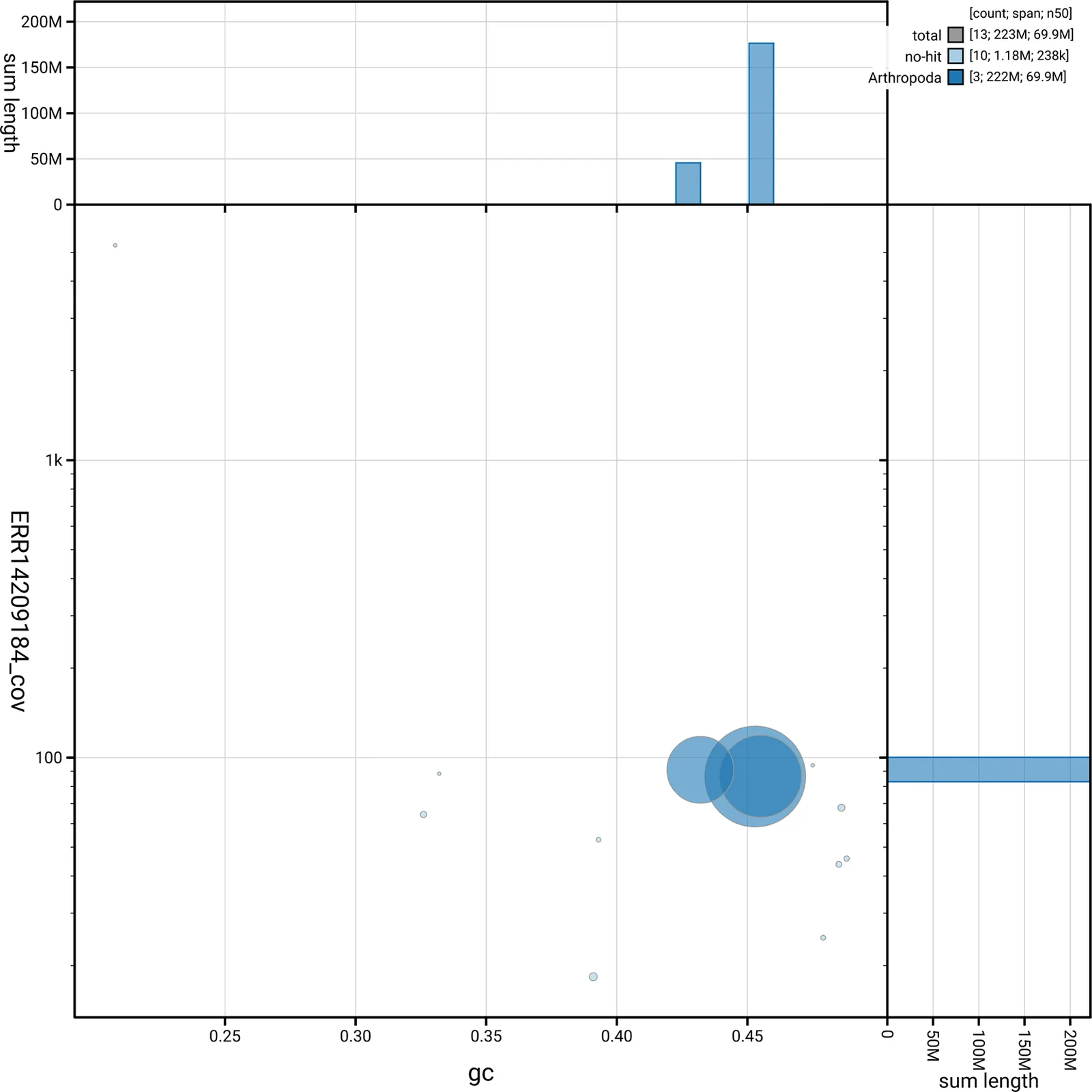
BlobToolKit blob plot for idGeoTrip2.1. The plot shows base coverage (vertical axis) and GC content (horizontal axis). The circles represent scaffolds, with the size proportional to scaffold length and the colour representing phylum membership. The histograms along the axes display the total length of sequences distributed across different levels of coverage and GC content. An interactive version of this figure is available on the BlobToolKit viewer.

BUSCO v.5.7.1 analysis using the diptera_odb10 reference set ( *n* = 3 285) identified 97.8% of the expected gene set (single = 97.0%, duplicated = 0.9%). The snail plot in Figure 5 summarises the scaffold length distribution and other assembly statistics for the primary assembly. The blob plot in Figure 6 shows the distribution of scaffolds by GC proportion and coverage.

Table 4 lists the assembly metric benchmarks adapted from Rhie *et al.* (2021) and the Earth BioGenome Project Report on Assembly Standards. The EBP metric, calculated for the primary assembly, is **6.C.Q65**, meeting the recommended 6.C.Q40 reference standard.

**Table 4.** Earth Biogenome Project summary metrics for the *Geomyza tripunctata* assembly.

Measure	Value	Benchmark
EBP summary (primary)	6.C.Q65	6.C.Q40
Contig N50 length	3.58 Mb	≥ 1 Mb
Scaffold N50 length	69.93 Mb	= chromosome N50
Consensus quality (QV)	Primary: 65.5; alternate: 66.1; combined: 65.9	≥ 40
*k*-mer completeness	Primary: 82.85%; alternate: 82.80%; combined: 99.17%	≥ 95%
BUSCO	C:97.8% [S:97.0%, D:0.9%], F:0.6%, M:1.6%, n:3 285	S > 90%; D < 5%
Percentage of assembly assigned to chromosomes	99.67%	≥ 90%

*Note:* The EBP summary uses log10(Contig N50); chromosome-level (C) or log10(Scaffold N50); Q (Merqury QV). BUSCO: C = complete; S = single-copy; D = duplicated; F = fragmented; M = missing; n = orthologues.

## Author information

Contributors are listed at the following links:

• Members of the University of Oxford and Wytham Woods Genome Acquisition Lab
• Members of the Darwin Tree of Life Barcoding collective
• Members of the Wellcome Sanger Institute Tree of Life Management, Samples and Laboratory team
• Members of Wellcome Sanger Institute Scientific Operations – Sequencing Operations
• Members of the Wellcome Sanger Institute Tree of Life Core Informatics team
• Members of the Tree of Life Core Informatics collective
• Members of the Darwin Tree of Life Consortium

## Wellcome Sanger Institute – Legal and Governance

The materials that have contributed to this genome note have been supplied by a Darwin Tree of Life Partner. The submission of materials by a Darwin Tree of Life Partner is subject to the **‘Darwin Tree of Life Project Sampling Code of Practice’**, which can be found in full on the Darwin Tree of Life website. By agreeing with and signing up to the Sampling Code of Practice, the Darwin Tree of Life Partner agrees they will meet the legal and ethical requirements and standards set out within this document in respect of all samples acquired for, and supplied to, the Darwin Tree of Life Project. Further, the Wellcome Sanger Institute employs a process whereby due diligence is carried out proportionate to the nature of the materials themselves, and the circumstances under which they have been/are to be collected and provided for use. The purpose of this is to address and mitigate any potential legal and/or ethical implications of receipt and use of the materials as part of the research project, and to ensure that in doing so we align with best practice wherever possible. The overarching areas of consideration are:

• Ethical review of provenance and sourcing of the material
• Legality of collection, transfer and use (national and international)

Each transfer of samples is further undertaken according to a Research Collaboration Agreement or Material Transfer Agreement entered into by the Darwin Tree of Life Partner, Genome Research Limited (operating as the Wellcome Sanger Institute), and in some circumstances, other Darwin Tree of Life collaborators.

## Data Availability

European Nucleotide Archive: Geomyza tripunctata. Accession number PRJEB85202; https://identifiers.org/ena.embl/PRJEB85202. The genome sequence is released openly for reuse. The *Geomyza tripunctata* genome sequencing initiative is part of the Darwin Tree of Life Project (PRJEB40665) and the Sanger Institute Tree of Life Programme (PRJEB43745). All raw sequence data and the assembly have been deposited in INSDC databases. The genome will be annotated using available RNA-Seq data and presented through the Ensembl pipeline at the European Bioinformatics Institute. Raw data and assembly accession identifiers are reported in Tables 1 and 2. Production code used in genome assembly at the WSI Tree of Life is available at https://github.com/sanger-tol . Table 5 lists software versions used in this study.
