# Supplementary material for: The genome sequence of an opomyzid fly, Geomyza tripunctata Fallen, 1823 (Diptera: Opomyzidae)
Source: Wellcome Open Res. 2026 Jul 20;11:453. [Version 1] doi: 10.12688/wellcomeopenres.27071.1 (PMC13570115; doi:10.12688/wellcomeopenres.27071.1)
Supplement: Peer review history

# Reviewer report: Reviewer response for version 1

**DOI:** 10.21956/wellcomeopenres.29797.r163750

**Published:** 2026-09-11

**Authors:** Jacob Wenger

**Affiliations:** 1 California State University, Fresno, USA

The authors have submitted a genome assembly for the opomyzid fly *Geomyza tripunctata*, representing the first such assembly within the genus.  The work was performed as part of the Darwin Tree of Life project, documenting the eukaryotic diversity of Britain & Ireland, though the species is also known in North America.  

 The methods and materials are all well described and clear, following publicly available Darwin Tree of Life protocols. PacBio HiFi long reads were combined with Hi-C sequencing to produce a highly contiguous 223.3MB assembly spread across three chromosome scaffolds.  Assembly size, completeness, continuity, and gene frequency are in line with what is expected in an insect genome. The authors are upfront with limitations of the paper (such as the lack of resolution of sex chromosomes).  

 Overall, this is a strong assembly, and a well written paper. 

 On minor comment:  In the final paragraph of the background section, it is noted that the genome was sequenced from a single male (SAMEA114655871). However, in the abstract and methods section the two sequenced adult flies are both listed as female. Perhaps this is a typographical error in the background?

Are sufficient details of methods and materials provided to allow replication by others?

Yes

Is the rationale for creating the dataset(s) clearly described?

Yes

Are the datasets clearly presented in a useable and accessible format?

Yes

Are the protocols appropriate and is the work technically sound?

Yes

Reviewer Expertise:

Entomology, genetics, genomics.

I confirm that I have read this submission and believe that I have an appropriate level of expertise to confirm that it is of an acceptable scientific standard.

# Reviewer report: Reviewer response for version 1

**DOI:** 10.21956/wellcomeopenres.29797.r161796

**Published:** 2026-07-24

**Authors:** Luis Diambra

**Affiliations:** 1 Univeridad Nacional de La Plata, La Plata, Argentina

This manuscript from Crowley et al. reports the high-quality genome assembly of the fly *Geomyza tripunctata*, as part of the Darwin Tree of Life project. The assembly was generated using PacBio HiFi reads (~84x coverage) and scaffolded into 3 chromosomal pseudomolecules using Hi-C data. Manual curation was performed using HiGlass, and assembly metrics were evaluated using BUSCO and BlobToolKit. The paper is well-structured and clear, following standard Darwin Tree of Life protocols. The assembled genome has exceptional continuity and completeness. In fact, the assembly achieved an N50 scaffold length of ~70 Mb, with 99.67% of the assembly scaffolded into 3 chromosomes. Further, BUSCO completeness is high at 97.8% with minimal duplication. Also, the consensus quality score (QV) of 65.5 far exceeds standard reference benchmarks. 

 The authors rightly note that sex chromosomes could not be definitively identified. Since the sequenced specimen was female (XX) and Diptera exhibit high sex chromosome turnover, sex chromosomes could not be reliably inferred by homology. 

 Overall, this is a solid, high-value data note that provides an excellent genomic resource for the field. The manuscript meets the standards of the journal and is ready for indexing.

Are sufficient details of methods and materials provided to allow replication by others?

Yes

Is the rationale for creating the dataset(s) clearly described?

Yes

Are the datasets clearly presented in a useable and accessible format?

Yes

Are the protocols appropriate and is the work technically sound?

Yes

Reviewer Expertise:

Bioinformatics

I confirm that I have read this submission and believe that I have an appropriate level of expertise to confirm that it is of an acceptable scientific standard.
